# Effects of Repeated Central Administration of Endothelin Type A Receptor Antagonist on the Development of Neuropathic Pain in Rats

**DOI:** 10.1155/2013/529871

**Published:** 2013-08-29

**Authors:** Lydia W. Tai, Victor K. L. Hung, Wei Mei, Qiu Qiu, Sookja K. Chung, C. W. Cheung

**Affiliations:** ^1^Department of Anaesthesiology, The University of Hong Kong, Queen Mary Hospital, Room 424, 4/F, Block K, 102 Pokfulam, Pokfulam, Hong Kong; ^2^Laboratory and Clinical Research Institute for Pain, The University of Hong Kong, Queen Mary Hospital, Room 424, 4/F, Block K, 102 Pokfulam, Pokfulam, Hong Kong; ^3^Department of Anaesthesiology, Tongji Hospital, Huazhong University of Science and Technology, Wuhan, China; ^4^Department of Anatomy, The University of Hong Kong, 1/F, Laboratory Block, Faculty of Medicine Building, 21 Sassoon Road, Hong Kong; ^5^Research Center of Heart, Brain, Hormone and Healthy Aging, The University of Hong Kong, 2/F, William MW Mong Block, Faculty of Medicine Building, 21 Sassoon Road, Pokfulam, Hong Kong

## Abstract

Endothelin-1 (ET-1) predominates in the endothelin family effectively in vascular tone control, mitogenesis, and neuromodulation. Its receptors are widespread in the central nervous system (CNS) associated with endogenous pain control, suggesting an important role of ET-1 in central pain processing. This study aimed to evaluate the effect of central ET-1 on the development of neuropathic pain behaviour by repeated intrathecal administration of endothelin type A receptor (ETAR) antagonist (BQ-123) in a sciatic nerve ligation (SNL) animal model. BQ-123 was administered intrathecally to rats at dosages 15 **μ**g, 20 **μ**g, 25 **μ**g, and 30 **μ**g, daily for 3 days. Mechanical allodynia was assessed daily 30 minutes before/after injection, 1 hour after injection of BQ-123 from post-SNL day 4 to day 6, and once on day 7 (without BQ-123 administration) before rats were sacrificed. Increasing trends of mechanical threshold were observed, and they reached significance at all dosages on post-SNL day 7 (*P* < 0.05 at dosage 15 **μ**g and *P* < 0.001 at dosages 20 **μ**g, 25 **μ**g, and 30 **μ**g) in comparison to control group. BQ-123 at dosage 30 **μ**g showed the most stable and significant mechanical threshold rise. Repeated central administration of BQ-123 alleviated mechanical allodynia after SNL. Our results provide insight into the therapeutic strategies, including timing, against neuropathic pain development with ETAR antagonist.

## 1. Introduction

The endothelin family majors in control of vascular tone and is also involved in a large variety of pain processes as a neuromodulator. It consists of three peptides: ET-1, endothelin-2 (ET-2), and endothelin-3 (ET-3) and two major receptor subtypes: endothelin type A receptor (ETAR) and endothelin type B receptor (ETBR). ET-1 is the predominant and most potent one among the three peptides and has higher binding affinity towards the ETAR. ET-1 and its two receptor subtypes are expressed at all levels of the nervous system [1, 2]. It is a neurotransmitter/modulator in addition to its vasoactive function in all mammalian species [3, 4]. The fact that endothelin receptors are widespread in the areas associated with endogenous pain control suggests that ET-1 may play a role in cortical pain processing [5]. In previous studies, ET-1 has been shown to be antinociceptive in the central nervous system (CNS) in contrast to its pronociceptive action in the periphery [6–9]. Direct application of ET-1 to the peripheral nervous system (PNS), such as subcutaneous injection, has been shown to elicit spontaneous pain or hyperalgesia in both animals and humans [10, 11], and local intervention with its receptor antagonists can abolish the overt nociceptive behaviour [12, 13]. In contrast, intrathecal administration of ET-1 was shown to suppress formalin-induced flinching behaviour in rats [14]. In a thermal pain model, the anti-nociceptive action of centrally injected ET-1 was blocked by an ETAR antagonist which suggests that antinociception of ET-1 in the CNS is mediated via ETAR [8]. 

Nevertheless, the effect of endogenous central ET-1 in the development of neuropathic pain remains unclear. Although most of the studies that have been done so far showed an analgesic effect of ET-1 in the CNS, it is unknown how endogenous ET-1 contributes to neuropathic pain because the above-mentioned studies were done using either animal inflammatory pain models or thermal pain tests to study the pain response after central administration of ET-1 with or without its receptor antagonists [14, 15]; moreover, exogenously injected ET-1 is an extra boost to the CNS that its effect may not represent the influence of endogenous level of ET-1 under pathological condition. In fact, there were only a few studies that related ET-1 to neuropathic pain, but they focused on periphery which demonstrated antiallodynic effects of ETAR/ETBR antagonists in the PNS [16, 17]. Thus, it would be interesting to test whether exogenous administration of only ET-1 receptor antagonists in the CNS would have analgesic effect in neuropathic pain or not. Since the contribution of central endogenous ET-1 to the development of neuropathic pain has not been adequately studied, we aim to investigate this subject by examining the effect of repeated intrathecal administration of an ETAR antagonist, BQ-123, on sciatic nerve ligation- (SNL-) induced neuropathic pain.

## 2. Materials and Methods

### 2.1. Animals

Animal experiments were conducted according to the US National Institute of Health Guide for the Care and Use of Laboratory Animals and were approved by the Committee on the Use of Live Animals in Teaching and Research from The University of Hong Kong. Adult male Sprague-Dawley (SD) rats (250~300 g) were used in this experiment. Rats were kept individually in plastic cages with a floor covered with soft bedding at room temperature and were maintained on a light/dark cycle of 12 hours day/night. Food and water were provided ad libitum. 

### 2.2. Sciatic Nerve Ligation Model

A sciatic nerve ligation (SNL) model using male, adult, SD rats was adopted in this study. The surgical technique for the right sciatic nerve was performed according to the method described by Seltzer et al. [18]. Animals were anesthetized with sodium pentobarbital 70 mg/kg administrated intraperitoneally. Under aseptic condition, an incision from the right sciatic notch to the distal thigh was made after the animal lost response to tail and toe pinch. After exposure of the sciatic nerve, a ligature about one-third to one-half the diameter of the sciatic nerve was made to the nerve with 7–0 silicon-treated silk suture. The wound was then closed with 2 to 3 skin sutures (4–0 cotton). In sham-operated animals, the sciatic nerve was exposed in the same manner but not ligated. 

### 2.3. Catheter Implantation

After SNL surgery, catheter implantation was performed. Polyethylene tube PE-10 (Clay Adams, USA) was implanted using a lumbar approach according to the method described [19]. An incision was made along the spinal cord at the pelvic girdle region, and a saline-filled sterile PE-10 tube catheter was inserted into the intervertebral space between vertebrae L5 and L6. The inserted part of the catheter was about 2 cm so it could reach the lumbar enlargement of the spinal cord. Correct intrathecal insertion was noted by a tail-flick or a twitch in the hind paw. Then the catheter was tunnelled rostrally under skin with a guide-cannula towards the occipital region to allow about 4 cm catheter appear to in the region for drug infusion. Ten microlitres of sterile saline was injected to flush and fill the catheter, and the end of the indwelling catheter was then sealed by melting it. The wound was closed with 3 to 4 sutures (4–0 cotton). The animals were then allowed to recover for 3 days after the surgery. Any rats with neurologic deficit due to the surgical procedure were excluded from the experiment. 

### 2.4. Study Drug BQ-123 Administration

BQ-123 (Calbiochem, USA) at four dosages 15 *μ*g, 20 *μ*g, 25 *μ*g, and 30 *μ*g were each dissolved in sterile saline and administrated intrathecally in a bolus of 10 *μ*L based on its usage in other pain-related studies [20, 21]. Animals were randomly assigned into six study groups as follows: (1) Sham group:Sham-operated (no ligature placement on sciatic nerve) animals treated with sterile saline (*n* = 6); (2) Control group: SNL-operated animals treated with sterile saline (*n* = 6); (3) Four BQ-123 drug groups: each group of SNL-operated animals were assigned to receive one of the four dosages 15 *μ*g, 20 *μ*g, 25 *μ*g, and 30 *μ*g (*n* = 6). 

### 2.5. Mechanical Allodynia Assessment

Mechanical threshold was assessed by testing the right hind paw withdrawal response to Von Frey probe of the Electrovonfrey apparatus (IITC/Life Science, Inc., USA). A transparent plastic dome with a metal mesh floor was used to hold the animal during assessment. Each animal was placed in the dome to allow access to the plantar surface of the hind paw through the metal mesh floor and allowed to accommodate to the environment for 30 minutes before testing. During the test, a Von Frey probe was pressed perpendicularly against the plantar surface of the hind paw with continuous force. A positive response was considered if the animal withdrew its hind paw within 6 to 8 seconds except any hind paw movements due to locomotion. If no response was observed, a stiffer probe was used until a response was observed. The force (in grams) to elicit such positive response was shown on the screen of the Electrovonfrey apparatus and recorded. The range of probe we used in this study was from 10 g to 17 g according to suggestions from a previous study [22]. The test was repeated three times for each rat at approximately 5-minute intervals, and the mean values of the three repetitions were counted as the mechanical threshold. The behavioural data were collected before surgery on day 0 (surgery day) for baseline and at three time points each day from day 4 to day 6: 30 minutes before injection, 30 minutes after injection, and 1 hour after injection. Only a single time point, before injection, was collected on day 7 as no drug was administered. All animals were sacrificed on day 7 after SNL after behaviour test. 

### 2.6. Statistics

All behavioural data were presented as mean ± S.E.M. One-way analysis of variance (ANOVA) with Tukey's multiple comparison posttest was used for comparison of different groups at one time point. Two-way ANOVA with Bonferroni multiple comparisons posttest was used for comparison between different groups at multiple time points. *P* value of <0.05 was considered significant. 

## 3. Results

**Figure 1:**
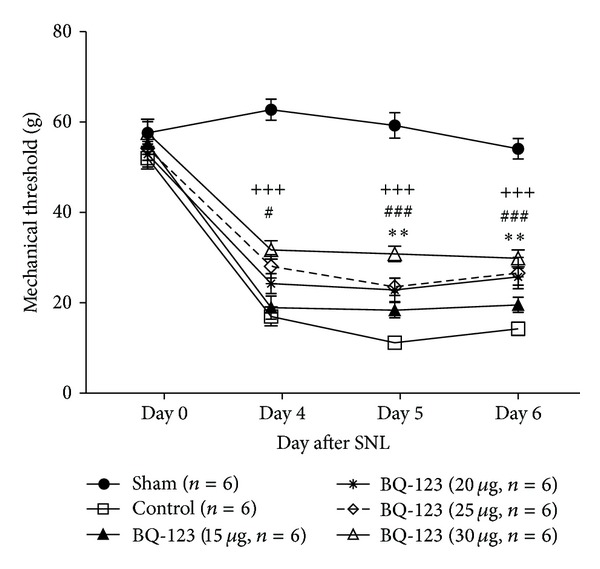
Effect of BQ-123 (15 *μ*g, 20 *μ*g, 25 *μ*g, and 30 *μ*g) on mechanical allodynia induced by sciatic nerve ligation (SNL) at 30 minutes after intrathecal administration. Drug administration started on day 4 and continued to day 6 after SNL for 3 consecutive days. Data presented as mean ± S.E.M and *n* = 6/group. Significance is presented as **P < 0.01 for BQ-123 20 *μ*g group versus control group; ^#^ *P* < 0.05 and ^###^ *P* < 0.001 for BQ-123 25 *μ*g group versus control group; ^+++^ *P* < 0.001 for BQ-123 30 *μ*g group versus control group.

**Figure 2:**
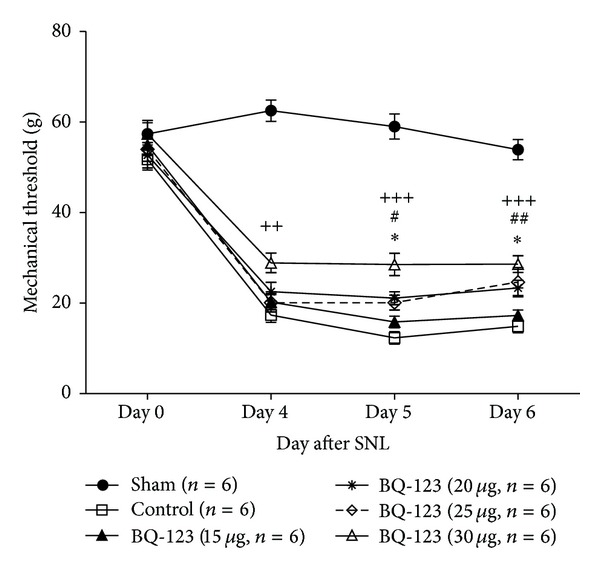
Comparison of mechanical threshold between BQ-123 treatment groups (15 *μ*g, 20 *μ*g, 25 *μ*g, and 30 *μ*g) and control group at 1 hour after intrathecal administration. Drug administration initiated on day 4 and continued to day 6 after sciatic nerve ligation (SNL) for 3 consecutive days. Data are presented as mean ± S.E.M and *n* = 6/group. Significance is presented as *P < 0.05 for BQ-123 20 *μ*g group versus control group; ^#^ *P* < 0.05 and ^##^ *P* < 0.01 for BQ-123 25 *μ*g group versus control group; ^++^ *P* < 0.01 and ^+++^ *P* < 0.001 for BQ-123 30 *μ*g group versus control group.

**Figure 3:**
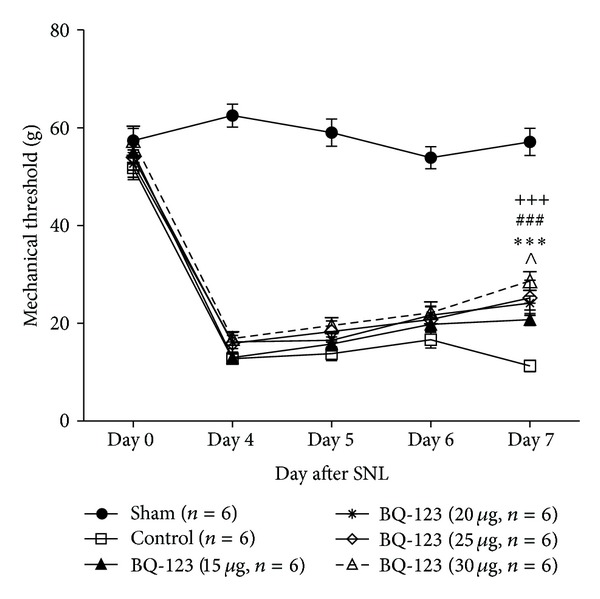
Comparison of mechanical threshold between BQ-123 treatment groups (15 *μ*g, 20 *μ*g, 25 *μ*g, and 30 *μ*g) and control group 30 minutes before intrathecal administration. Assessment was made on day 7 that no drug was administered. Data presented as mean ± S.E.M and *n* = 6/group. Significance is presented as ^∧^ *P* < 0.05 for BQ-123 15 *μ*g group versus control group; ***P < 0.001 for BQ-123 20 *μ*g group versus control group; ^###^ *P* < 0.001 for BQ-123 25 *μ*g group versus control group; ^+++^ *P* < 0.001 for BQ-123 30 *μ*g group versus control group. SNL: sciatic nerve ligation.

The present study examined the effect of ETAR antagonist in the CNS on the development of SNL-induced neuropathic pain by treating SNL-operated rat with a daily intrathecal injection of BQ-123 at days 4, 5, and 6 after surgery. Administration dosage of BQ-123 ranged between 15 *μ*g, 20 *μ*g, 25 *μ*g, and 30 *μ*g. We recorded mechanical thresholds of animals at 30 minutes (Figure 1) or 1 hour (Figure 2) after administration of BQ-123 or 30 minutes prior to administration at post-SNL days 4, 5, and 6 (Figure 3) as well as day 7 which was 24 hours after the last injection of BQ-123 (Figure 3). We observed significant decrease in mechanical thresholds in all SNL-operated groups (control and drug groups) when compared to sham group throughout the time course of experiment (Figures 1, 2, and 3, all *P* < 0.001). Sham group did not show any significant change of mechanical threshold to Von Frey stimulation throughout the study. After intrathecal administration of BQ-123, elevated trends of mechanical thresholds were seen in all BQ-123 drug groups as shown in Figures 1, 2, and 3.

### 3.1. BQ-123 Group at Dose of 15 *μ*g

BQ-123 at dose of 15 *μ*g induced slight elevation of threshold when compared to the control group 30 minutes and 1 hour after intrathecal administration of BQ-123 on day 4 to day 6, but the results were not statistically significant (Figures 1 and 2). Significant anti-allodynic effect was observed on post-SNL day 7 compared with control group (Figure 3, *P* < 0.05).

### 3.2. BQ-123 Group at Dose of 20 *μ*g

BQ-123 at dose of 20 *μ*g produced significant antiallodynic effect both at 30 minutes and 1 hour after intrathecal drug administration. On days 5 and 6, significant increase of mechanical threshold was observed at 30 minutes (Figure 1, *P* < 0.01) and 1 hour (Figure 2, *P* < 0.05) after administration, compared with control group, respectively. From mechanical threshold examined before daily administration on post-SNL day 4 to day 6, gradual increase was observed, and it was significant on post-SNL day 7 (Figure 3, *P* < 0.001) when compared with control group.

### 3.3. BQ-123 Group at Dose of 25 *μ*g

Higher trend of elevation of mechanical threshold was demonstrated at dose of 25 *μ*g throughout the time course of experiment, compared with that at doses of 15 *μ*g and 20 *μ*g. On day 4, significant anti-allodynic effect was observed at 30 minutes (Figure 1, *P* < 0.05) but not 1 hour (Figure 2) after intrathecal drug administration when compared with control group. On day 5, BQ-123 produced significant increase in mechanical threshold at both time points of 30 minutes (Figure 1, *P* < 0.001) and 1 hour (Figure 2, *P* < 0.05) after drug administration, compared with control group. Similar trends were also observed on day 6 where significance of *P* < 0.001 and *P* < 0.01 was found, at 30 minutes and 1 hour after intrathecal administration compared to control group, respectively (Figures 1 and 2). The antiallodynic effect of BQ-123 on post-SNL day 7 was shown in Figure 3, and the effect was significant compared to control group (*P* < 0.001). 

### 3.4. BQ-123 Group at Dose of 30 *μ*g

BQ-123 at dose of 30 *μ*g achieved the most significant effect in amelioration of mechanical allodynia throughout the study period. Significant elevation of mechanical threshold was shown from day 4 to day 6 at 30 minutes after drug administration compared to control group (Figure 1, all *P* < 0.001). In Figure 2, anti-allodynic effect was shown to remain significant 1 hour after drug administration on day 4 (*P* < 0.01) and days 5 and 6 (both *P* < 0.001) compared to control group. At the preinjection time points, BQ-123 at dose of 30 *μ*g showed the greatest increase in mechanical threshold among other dosages from post-SNL day 5. The threshold peaked significantly on post-SNL day 7 when compared with control group (Figure 3, *P* < 0.001). 

## 4. Discussion

Our results showed that daily intrathecal injections of ETAR antagonist BQ-123 for 3 consecutive days from day 4 to day 6 after SNL alleviated mechanical allodynia with maximum effect achieved at dose of 30 *μ*g in SNL neuropathic pain model. To date, this is the first study to examine the effect of ETAR antagonist in the CNS on the development of SNL-induced neuropathic pain. 

The SNL model is currently one of the classic neuropathic pain models that produces spontaneous hind paw nociception, hyperalgesia, and pronounced mechanical allodynia [18]. Previous studies using this model showed that mechanical threshold reached the lowest point about a week after surgery and remained stable for up to 20 weeks [22–27]. Therefore, to investigate the influence of ETAR antagonist in the CNS on the development of mechanical allodynia, one of the major symptoms of neuropathic pain, we evaluated animal's mechanical withdrawal threshold within a week after SNL. 

ET-1 has been suggested to modulate pathological pain processing by the differential regulation of its receptors—ETAR or ETBR [28–30]. The role of ET-1 and its receptors in nociception and pathological pain has been described extensively in animal studies following the direct administration of exogenous ET-1 peptide with or without pharmacological interventions on ET receptor activities [13–17, 31–33]. However, only few studies of ET-1 in neuropathic pain were done in intact animal models which all focused in periphery [30]. Amongst these studies, one pointed out that ETAR was mainly involved in neuropathic pain at the injury site induced by chronic constriction injury (CCI) of sciatic nerve [17]. As central sensitization is a major mechanism involved in the development of neuropathic pain, therefore we were interested in exploring the effect of endogenous central ET-1 on SNL-induced neuropathic pain. Although ET-1 has been shown to be analgesic in the CNS when administered in a bolus [34], this result may not indicate the effect of innate ET-1 in pain processing because the exogenous dose is an extra boost to the CNS that it is abnormal to the system. In fact, a previous study showed that systemic administration of ETAR antagonist attenuated tactile allodynia in diabetic neuropathic pain, suggesting that the interaction between endogenous ET-1 and ETAR contributes to pain sensitization [16]. Therefore, by only antagonizing ETAR in the CNS, we may know how endogenous ET-1 is involved in central pain processing during the development of neuropathic pain. From the BQ-123 groups with different dosages (15 *μ*g, 20 *μ*g, 25 *μ*g, and 30 *μ*g), we found that drug group with higher dosage showed higher trend of elevation in mechanical threshold. Through the time course of tactile sensitivity changes in animals after treatment of BQ-123, we observed similar elevated trends in mechanical threshold at both 30 minutes and 1 hour following injection, showing dose-dependent tendency. The present result implies that normal interactions between ET-1 and ETAR contribute to pain sensitization in neuropathic pain. This provides a new insight into the current thoughts that ET-1 is antinociceptive in the CNS. We speculate that the level of ET-1 in the CNS may affect its actions on its receptors differently in pain modulation because the injected bolus of ET-1 produced analgesic effect as shown in previous studies [8, 14] while the endogenous level of ET-1 in the CNS did not exhibit such effect under pathological condition in our study because ETAR antagonist reduced pain sensation.

The effect of the drug also seemed to accumulate slowly over the 3 days of once-per-day drug administration because the mechanical threshold of the drug groups started to increase and peaked on post-SNL day 7. This evidence supports the involvement of ET-1 in central sensitization as suggested by a previous study [35], and this action is mediated possibly via ETAR since prior exposure to its blockage increased mechanical threshold gradually. This gradual rise in mechanical threshold was noticeable every day after prior-day exposure, but the change was not significant until day 7 which can be due to the fact that the mechanical threshold of the drug groups reached its highest point while that of the control group was further decreased on that day. This observation implies that repeated blockage of ETAR improves the progression of the development of neuropathic pain. Taken together, present results provide a new aspect that repeated central administration of ETAR antagonist may be effective in improving mechanical allodynia during the development of neuropathic pain. 

In addition, since higher dosage produced more significant effect in alleviating mechanical allodynia, the result again supports our hypothesis that ET-1 modulates neuropathic pain development partially via ETAR in the CNS. In the present study, the highest BQ-123 concentration, 30 *μ*g, elicited the greatest increase in the threshold and produced the most significant improvement among the four given dosages throughout the period of experiment. Although the effects of the other three drug groups fluctuated at some time points, they still showed increasing trends and significant elevation of mechanical threshold near the end of the experiment days (post-SNL day 7). The given dosages in these drug groups were not enough to maintain a constant significant effect but were still able to elevate the mechanical pain threshold to a certain degree after repeated administration. Our result is similar to the situation in the periphery where ETAR antagonist was found to reverse mechanical allodynia while the ETBR antagonist did not [17]. These facts together suggest that ET-1 contributes to both peripheral and central sensitization via ETAR in neuropathic pain states. This is interesting compared to a documented mechanism of ET-1 where it was found to induce pain mainly through ETBR in the periphery and central inflammatory pain [12, 14, 36], suggesting that more than one mechanism is involved in modulation of central pain processing by ET-1. 

In conclusion, our study demonstrated that repeated central administration of BQ-123 could alleviate mechanical allodynia during the development of SNL-induced neuropathic pain. Higher dosage (30 *μ*g) of BQ-123 could also induce more stable and significant increase in pain threshold in SNL-induced neuropathic pain. These observed behaviour alterations contribute a new insight to the current roles of ET-1 in pain modulation and potentially provide important information for future studies in searching putative pharmacological intervention, including timing, for prevention and treatment of neuropathic pain.

## References

[1] Berti-Mattera LN, Gariepy CE, Burke RM, Hall AK (2006). Reduced expression of endothelin B receptors and mechanical hyperalgesia in experimental chronic diabetes. *Experimental Neurology*.

[2] Pomonis JD, Rogers SD, Peters CM, Ghilardi JR, Mantyh PW (2001). Expression and localization of endothelin receptors: implications for the involvement of peripheral glia in nociception. *Journal of Neuroscience*.

[3] Naidoo V, Naidoo S, Mahabeer R, Raidoo DM (2004). Cellular distribution of the endothelin system in the human brain. *Journal of Chemical Neuroanatomy*.

[4] Giaid A, Gibson SJ, Herrero MT (1991). Topographical localisation of endothelin mRNA and peptide immunoreactivity in neurones of the human brain. *Histochemistry*.

[5] Kurokawa K, Yamada H, Ochi J (1997). Topographical distribution of neurons containing endothelin type A receptor in the rat brain. *The Journal of Comparative Neurology*.

[6] Davar G, Hans G, Fareed MU, Sinnott C, Strichartz G (1998). Behavioral signs of acute pain produced by application of endothelin-1 to rat sciatic nerve. *NeuroReport*.

[7] Motta EM, Chichorro JG, D’Orléans-Juste P, Rae GA (2009). Roles of endothelin ETA and ETB receptors in nociception and chemical, thermal and mechanical hyperalgesia induced by endothelin-1 in the rat hindpaw. *Peptides*.

[8] Hasue F, Kuwaki T, Yamada H, Fukuda Y, Shimoyama M (2004). Inhibitory actions of endothelin-1 on pain processing. *Journal of Cardiovascular Pharmacology*.

[9] D’Amico M, Berrino L, Maione S, Filippelli A, de Novellis V, Rossi F (1996). Endothelin-1 in periaqueductal gray area of mice induces analgesia via glutamatergic receptors. *Pain*.

[10] Gokin AP, Fareed MU, Pan H, Hans G, Strichartz GR, Davar G (2001). Local injection of endothelin-1 produces pain-like behavior and excitation of nociceptors in rats. *Journal of Neuroscience*.

[11] Raffa RB, Schupsky JJ, Jacoby HI (1996). Endothelin-induced nociception in mice: mediation by ETA and ETB receptors. *Journal of Pharmacology and Experimental Therapeutics*.

[12] Baamonde A, Lastra A, Villazón M, Bordallo J, Hidalgo A, Menéndez L (2004). Involvement of endogenous endothelins in thermal and mechanical inflammatory hyperalgesia in mice. *Naunyn-Schmiedeberg’s Archives of Pharmacology*.

[13] Piovezan AP, D’Orléans-Juste P, Souza GEP, Rae GA (2000). Endothelin-1-induced ET(A) receptor-mediated nociception, hyperalgesia and oedema in the mouse hind-paw: modulation by simultaneous ET(B) receptor activation. *The British Journal of Pharmacology*.

[14] Yamamoto T, Shimoyama N, Asano H, Mizuguchi T (1994). Analysis of the role of endothelin-A and endothelin-B receptors on nociceptive information transmission in the spinal cord with FR139317, an endothelin-A receptor antagonist, and sarafotoxin S6c, an endothelin-B receptor agonist. *Journal of Pharmacology and Experimental Therapeutics*.

[15] Nikolov R, Maslarova J, Semkova I, Moyanova S (1992). Intracerebroventricular endothelin-1 (ET-1) produces Ca^2+^-mediated antinociception in mice. *Methods and Findings in Experimental and Clinical Pharmacology*.

[16] Jarvis MF, Wessale JL, Zhu CZ (2000). ABT-627, an endothelin ET(A) receptor-selective antagonist, attenuates tactile allodynia in a diabetic rat model of neuropathic pain. *European Journal of Pharmacology*.

[17] Klass M, Hord A, Wilcox M, Denson D, Csete M (2005). A role for endothelin in neuropathic pain after chronic constriction injury of the sciatic nerve. *Anesthesia and Analgesia*.

[18] Seltzer Z, Dubner R, Shir Y (1990). A novel behavioral model of neuropathic pain disorders produced in rats by partial sciatic nerve injury. *Pain*.

[19] Størkson RV, Kjørsvik A, Tjølsen A, Hole K (1996). Lumbar catheterization of the spinal subarachnoid space in the rat. *Journal of Neuroscience Methods*.

[20] Zhou QL, Strichartz G, Davar G (2001). Endothelin-1 activates ETA receptors to increase intracellular calcium in model sensory neurons. *NeuroReport*.

[21] D’Amico M, Berrino L, Maione S, Rossi F (1996). Selective and non-selective ET antagonists reveal an ETB receptors mediated ET-1-induced behavioural effect in conscious rats. *Life Sciences*.

[22] Bennett GJ, Chung JM, Honore M, Seltzer Z (2003). Models of neuropathic pain in the rat. *Current Protocols in Neuroscience*.

[23] Smits H, van Kleef M, Joosten EA (2012). Spinal cord stimulation of dorsal columns in a rat model of neuropathic pain: evidence for a segmental spinal mechanism of pain relief. *Pain*.

[24] Nirogi R, Goura V, Shanmuganathan D, Jayarajan P, Abraham R (2012). Comparison of manual and automated filaments for evaluation of neuropathic pain behavior in rats. *Journal of Pharmacological and Toxicological Methods*.

[25] Kuwahata H, Komatsu T, Katsuyama S (2013). Peripherally injected linalool and bergamot essential oil attenuate mechanical allodynia via inhibiting spinal ERK phosphorylation. *Pharmacology, Biochemistry, and Behavior*.

[26] Kiguchi N, Maeda T, Kobayashi Y, Fukazawa Y, Kishioka S (2010). Macrophage inflammatory protein-1*α* mediates the development of neuropathic pain following peripheral nerve injury through interleukin-1*β* up-regulation. *Pain*.

[27] Takahashi Y, Hasegawa-Moriyama M, Sakurai T, Inada E (2011). The macrophage-mediated effects of the peroxisome proliferator-activated receptor-*γ* agonist rosiglitazone attenuate tactile allodynia in the early phase of neuropathic pain development. *Anesthesia and Analgesia*.

[28] Hans G, Deseure K, Adriaensen H (2008). Endothelin-1-induced pain and hyperalgesia: a review of pathophysiology, clinical manifestations and future therapeutic options. *Neuropeptides*.

[29] Hans G, Schmidt BL, Strichartz G (2009). Nociceptive sensitization by endothelin-1. *Brain Research Reviews*.

[30] Khodorova A, Montmayeur JP, Strichartz G (2009). Endothelin receptors and pain. *Journal of Pain*.

[31] Khodorova A, Navarro B, Jouaville LS (2003). Endothelin-B receptor activation triggers an endogenous analgesic cascade at sites of peripheral injury. *Nature Medicine*.

[32] Khodorova A, Richter J, Vasko MR, Strichartz G (2009). Early and late contributions of glutamate and CGRP to mechanical sensitization by endothelin-1. *Journal of Pain*.

[33] Joseph EK, Gear RW, Levine JD (2011). Mechanical stimulation enhances endothelin-1 hyperalgesia. *Neuroscience*.

[34] Chen G, Tanabe K, Yanagidate F (2012). Intrathecal endothelin-1 has antinociceptive effects in rat model of postoperative pain. *European Journal of Pharmacology*.

[35] Khodorova A, Strichartz GR (2010). Contralateral paw sensitization following injection of endothelin-1: effects of local anesthetics differentiate peripheral and central processes. *Neuroscience*.

[36] da Cunha JM, Rae GA, Ferreira SH, Cunha FDQ (2004). Endothelins induce ETB receptor-mediated mechanical hypernociception in rat hindpaw: roles of cAMP and protein kinase C. *European Journal of Pharmacology*.

